# Integrative plasma-to-spatial proteomics reveals fibroblast-associated signatures in liver metastatic breast cancer

**DOI:** 10.1186/s12935-026-04416-x

**Published:** 2026-07-13

**Authors:** Elif Öcal, Janik Riese, Ursula Schneider, Mark P. Kühnel, Danny Jonigk, Heidrun Hirner-Eppeneder, Stefanie Corradini, Osman Öcal, S. Nahum Goldberg, Max Seidensticker, Jens Ricke, Moritz Wildgruber, Marianna Alunni-Fabbroni

**Affiliations:** 1 Department of Radiology, LMU University Hospital, LMU Munich, Munich, Germany; 2 Institute of Pathology, Aachen University Hospital, Aachen, Germany; 3 Center for Integrated Oncology Aachen Bonn Cologne Duesseldorf (CIO ABCD), Aachen, Germany; 4 Department of Radiation Oncology, University Hospital, LMU Munich, Munich, Germany; 5 Department of Diagnostic and Interventional Radiology, Heidelberg University Hospital, Heidelberg, Germany; 6 Department of Radiology, Hadassah Hebrew University Medical Center, Jerusalem, 12000 Israel; 7 Institute of Pathology, Heidelberg University Hospital, Im Neuenheimer Feld 224, 69120 Heidelberg, Germany

**Keywords:** Proteomics, Liquid biopsy, Digital spatial profiling, Metastatic breast cancer, Locoregional therapy, Cancer-associated fibroblasts

## Abstract

**Background:**

Liver metastases from breast cancer (BCLM) are associated with aggressive disease and poor survival outcomes. Liver-directed locoregional therapies (LRT) based on radiation are limited by resistance and relapse. While liquid biopsy enables systemic monitoring, it provides limited insight into the tumor microenvironment and mechanisms of resistance. In this hypothesis-generating study, high-throughput plasma proteomics alongside digital spatial profiling of matched tumor tissues was employed to link systemic signals with local tumor biology, aiming to elucidate mechanisms underlying therapy response.

**Methods:**

Plasma and tissue samples were collected from BCLM (*n* = 14) patients receiving LRT. High-throughput proteomic analysis (Olink Proteomics, Uppsala, Sweden) was run to detect and quantify circulating proteins in plasma. Matched tissue samples were obtained before LRT and analyzed using Digital Spatial Profiling (GeoMX, NanoString Technologies, Seattle, WA) to assess the protein expression within defined tissue compartments. Results were then correlated with clinical outcome.

**Results:**

Systemic proteomic analysis showed significant high levels of CD8A, CX3CL1, FGF-21 before treatment in non-survivors suggesting a hyperactivated yet ineffective immune state. In contrast, survivors exhibited elevated plasma MMP1 (*p* = 0.035, AUC = 0.896) alongside reduced intratumoral levels. Spatial analysis did not identify significant differences in the distribution of immune cell signaling within the TME. However, compartment-specific profiling illuminated critical patterns: Ki-67 in immune stroma, not tumor cells, correlated with response, while fibronectin and α-SMA were abnormally enriched in tumor epithelial cells of non-survivors. These findings were consistent with a mesenchymal-like signature associated with poor survival, highlighting epithelial fibronectin as a candidate biomarker that warrants further investigation.

**Conclusion:**

Integrating plasma and spatial proteomics reveals key resistance mechanisms to LRT in BCLM with the opposing roles of MMP1 and fibronectin in blood versus tumor. Cancer-associated fibroblasts, linked to the epithelial compartment of the tumor, may represent potential targets for future investigation, particularly through MMP1 inhibition, potentially acting as adjuvants to radiation therapy.

**Supplementary Information:**

The online version contains supplementary material available at 10.1186/s12935-026-04416-x.

## Background

Breast cancer (BC) is the most commonly diagnosed malignancy among women and the second leading cause of cancer-related death in the United States [1]. Mortality is primarily driven by distant metastases, most frequently involving the liver, lungs, brain, and bones [2, 3]. While the five-year overall survival rate for non-metastatic BC approaches 99%, it declines sharply to 29% in metastatic disease [4, 5]. In breast cancer with liver metastases (BCLM), median survival without treatment ranges from 4 to 8 months [6], but therapeutic interventions can extend survival can extend up to three years in the oligometastatic settings [7–10]. In recent years, liver-directed, radiation-based, locoregional therapies (LRT) have gained prominence due to their favorable safety profile and ability to achieve durable local control [11–14]. Nevertheless, many patients experience recurrence or progression [15], partly reflecting the modulatory influence of the tumor microenvironment (TME), alongside other contributing mechanisms. The TME comprises a dynamic network of stromal cells, immune infiltrates, extracellular matrix components, and signaling molecules that collectively shape tumor behavior. In BCLM, the TME contributes to therapeutic resistance through mechanisms such as immune suppression [16, 17], dormancy reactivation induced by cancer-associated fibroblasts (CAFs) [18, 19], and dysregulated cytokine signaling [20, 21]. Elucidating these processes is therefore critical to understanding treatment resistance and disease progression. Proteomic analysis, both systemic and tissue-based, offers a comprehensive characterization of the proteins driving TME dynamics and therapeutic resistance [22]. In liquid biopsy, circulating plasma proteins reflect tumor-derived and immune-related components released into the bloodstream. High-throughput systemic proteomics thus enables minimally invasive, real-time profiling of molecular pathways active across the tumor-host interface. In tissue biopsies digital spatial profiling (DSP) provides high-plex, spatially resolved protein characterization, revealing molecular dynamics inaccessible through bulk analysis [23].

In this exploratory study, we integrated high-throughput plasma proteomic with DSP-based profiling of matched tumor tissues from BCLM patients treated with LRT. By bridging systemic and local proteomic landscapes, this approach aimed to uncover molecular signatures that may enhance the identification of predictive biomarkers and inform personalized therapeutic strategies.

## Methods

### Ethical considerations

This study represents a sub-analysis of all available liver metastatic breast cancer patients treated with radiation-based therapy at the Department of Radiology, LMU Hospital (German Clinical Trials Register-ID: DRKS00009744, DRKS00009916, DRKS00010587, DRKS00034084). All study protocols were conducted in accordance with the Declaration of Helsinki. Before entering the study, all patients were fully informed by the treating physician of the scope and goals of the trial, had given written informed consent, and were willing to comply with the study protocol.

### Study population and design

The sub-analysis described herein comprised patients diagnosed with breast cancer liver metastasis at the time of inclusion. Histological confirmation of malignancy was performed before therapy by percutaneous biopsy of liver lesions. Patients with metastatic breast cancer were treated by radiation-based loco-regional therapy (high-dose rate brachytherapy or selective internal radiation therapy) based on the extent of the disease [24]. According to dose conversion calculations, the radiation dose ranges delivered to the liver parenchyma were comparable when recalculated in terms of standard fractionation [25]. Follow-up imaging was performed at three months intervals using MRI and CT.

### Patient response assessment

Patient response to liver-directed therapy was assessed based on overall survival (OS) at 24 months post-treatment. A patient who remained alive at or beyond the 24-month follow-up was classified as *survivor*, while a patient who succumbed to disease within this period was categorized as *non-survivor*. This survival-based stratification was chosen to reflect meaningful long-term benefit from therapy, as 24-months survival in the context of BCLM is associated with significantly improved prognosis and may indicate a potentially durable response to locoregional therapy. This threshold is also in line with previously reported studies evaluating clinical outcomes in this patient population [26–30]. All clinical data were retrospectively reviewed, and survival status was confirmed through longitudinal medical record analysis. Survival analyses should be interpreted as hypothesis generating rather than powered for definitive inferential conclusions.

### Treatment modality distribution and survival outcomes

Patients underwent two different locoregional treatment modalities: high-dose rate brachytherapy (HDR-BT) and selective internal radiation therapy (SIRT). Among the 14 patients included in the study, 6 received HDR-BT and 8 were treated with SIRT. In the HDR-BT group, the majority of patients (*n* = 5, 83.3%) were classified as survivors at the 24-month follow-up, with only 1 (16.7%) non-survivor. In contrast, within the SIRT group, most patients (*n* = 7, 87.5%) were categorized as non-survivors, while only 1 (12.5%) patient met the criteria for long-term survival. This distribution highlights an imbalance in survival outcomes between the two treatment modalities, which should be interpreted cautiously given the limited cohort size.

### Sample collection

Peripheral blood (5mL) was collected at baseline (BL) and 48 h after procedure in Monovette EDTA tubes (Sarstedt AG, Nümbrecht, Germany). Centrifugation was performed within two hours at 3000 rpm for 5 min at 4 °C. Plasma aliquots were stored at − 80 °C until use. Tissue samples were obtained through an 18-gauge coaxial biopsy needle, fixed in 10% formalin overnight at 4 °C, and subsequently embedded in paraffin.

### Multiplex systemic proteomics

To characterize the protein composition of plasma samples, we employed high-throughput proteomic profiling using the Olink proteomics Target 96 Immuno-Oncology and Inflammation panels (Olink Proteomics, Uppsala, Sweden), based on Proximity Extension Assay (PEA) technology [31, 32], each measuring 92 protein biomarkers along with internal controls. The two panels were analyzed independently. Downstream analyses were conducted separately for each panel. Each analysis was conducted using 10µL of plasma. To minimize batch effects, samples were randomly distributed across a 96-well plate, with inter-plate controls included to correct for variations in between plates. Negative controls were also incorporated to define the limit of detection. Protein concentrations are reported in normalized protein expression (NPX) units on a log2 scale, with higher and lower NPX values indicating corresponding levels of protein concentration. The full list of quantified proteins is provided in Supplementary Tables 2 and 3.

### GeoMX digital spatial profiling

In order to perform proteomic analysis in tumor samples, Digital Spatial Profiling (DSP; NanoString Technologies, Seattle, WA) was performed on tissues collected prior to therapy. Serial 4 μm sections were cut from formalin-fixed, paraffin-embedded (FFPE) tumor tissues. Slides were dewaxed, rehydrated through graded alcohols, and subjected to heat-induced epitope retrieval using a citrate-based buffer (pH 6.0) at high temperature, according to manufacturer’s recommendations. Tissue sections were then incubated overnight at 4 °C with fluorescently labeled morphology markers: Pan-Cytokeratin (PanCK), CD45, and CD68. Syto13 was used to stain and identify cell nuclei. Segmentation based on cell-type markers identified areas enriched in epithelial tumor cells (panCK+/CD45−/CD68−) and immune-stromal regions (panCK−). The immune-stromal compartments were defined as panCK-negative regions containing immune and stromal elements. Within this compartment, macrophages (panCK−/CD45+/CD68+) were assessed separately from other leukocyte populations (panCK−/CD45+/CD68−). Thus, the immune-stromal compartment represents a broader stromal immune region that may include macrophage-enriched areas but is not restricted exclusively to macrophages [33]. The additional markers included in the panel, such as CD45 and CD68, were used to further characterize immune cell subpopulations (leukocytes and macrophages, respectively), rather than to define the primary segmentation. Thus, compartment delineation was based on PanCK expression, while immune markers were used for downstream phenotypic characterization. These markers were used to characterize immune cell infiltration within the stromal microenvironment. Regions of interest (ROI) were assessed by two independent pathologists. In total, spatial proteomic analyses were performed on 40 ROIs derived from 14 patients, resulting in 120 area of illumination (AOI) across epithelial and immune-stromal compartments. Following visualization staining, the tissue was incubated overnight at 4 °C with a multiplexed cocktail of 49 unique photocleavable oligonucleotide (PC-oligo) - conjugated primary antibodies. These antibodies targeted a broad range of proteins related to immune cell typing, immune activation status, drug targets, and myeloid cell markers (Table 1).

**Table 1:** Nanostring GeoMX human protein panels

Immune cell profiling	Immune cell typing	Immune activation status	IO Drug target	Myeloid
Ms IgG1	GZMB	CD127	4-1BB	CD11b
Ms IgG2a	CD20	CD25	B7-H3	CD11c
Rb IgG	CD3	CD27	GITR	CD14
GAPDH	CD34	CD44	IDO1	CD39
HistoneH3	CD4	CD45RO	OX40L	CD40
S6	CD56	CD80	STING	ARG1
β2-Microglobulin	CD66b	ICOS	CTLA4	CD163
CD31	CD8	PD-1	LAG3	CD68
CD45	FOXP3	PD-L1	Tim-3	HLA-DR
Ki-67	Fibronectin	PD-L2	VISTA	

Each AOI was sequentially illuminated with UV light using the GeoMx DSP instrument, allowing segmentation-specific photocleavage of the oligonucleotide tags from the bound antibodies within the defined AOIs. The released oligonucleotides were collected via a microcapillary system and transferred to individual wells of a 96-well plate. Collected oligonucleotides were then hybridized to fluorescent, 4-color, 6-spot barcodes and digitally counted using the nCounter Analysis System (NanoString Technologies). Raw counts for each protein target were first normalized to internal spike-in controls, followed by normalization to the geometric mean of two housekeeping proteins, GAPDH and ribosomal protein S6, within each AOI.

### Validation of proteomic signatures via immunohistochemistry

To validate the proteomics results obtained from plasma samples, we performed tissue immunohistochemistry to identify the level of expression of matrix metallopeptidase 1 (MMP1) and fibroblast activation protein (FAP). Two µm-thick sections were cut from formalin-fixed, paraffin-embedded tumor tissues, dewaxed, and rehydrated according to standard procedure (preheating at 60 C°; deparaffinization in Neo-Clear, Merck KGaA, Darmstadt, Germany; rehydration in graded series of ethanol and distilled water). Antigen retrieval was performed in Universal HIER Antigen Retrieval Reagent (Abcam, Cambridge, UK) by heating in a microwave for 20 min. For IHC analysis, the primary antibody (anti-MMP1, rabbit IgG polyclonal, Abcam, Cambridge, UK; Catalog ab52631, dilution 1:50 in SignalStain Antibody diluent, Cell Signaling Technology, Danvers MA; anti-FAP, rabbit IgG polyclonal, Invitrogen/ThermoFisher Scientific, Carlsbad CA, USA; Catalog PA5-99458, dilution 1:50 in SignalStain Antibody diluent, Cell Signaling Technology) was applied overnight at 4 C°, followed by incubation with secondary antibody (goat anti-rabbit IgG, Abcam; catalog ab205718, dilution 1:200 in SignalStain Antibody diluent, Cell Signaling Technology). DAB substrate kit (DAB Substrate Kit; Cell Signaling Technology) was used as chromogen. Counterstaining was performed with Haemalaun (Merck KGaA). For morphological reference, adjacent sections were stained with hematoxylin and eosin (H&E). After dehydration in graded sections of ethanol and Neo-Clear (Merck KGaA), slides were mounted with Neo-Mount (Merck KGaA).

### Statistical analysis

For comparisons between two groups, normally distributed variables were analyzed using paired or unpaired t-tests, while non-normally distributed variables were compared using the Mann-Whitney U test. Correlation analyses were performed using Pearson’s correlation. Normality of the variables was assessed using the Shapiro-Wilk test. Receiver operating characteristic (ROC) curve analysis identified optimal protein cut-off values that maximized sensitivity and specificity for predicting individual survival. Overall survival was estimated using the Kaplan-Meier method, and statistical significance was evaluated using the log-rank test. Due to the small sample size and imbalance between treatment groups, no formal subgroup or adjusted survival analyses by treatment modality were performed. These analyses are therefore to be interpreted as exploratory and independent of treatment type. Univariate Cox proportional hazards regression analysis was conducted to evaluate the association between candidate biomarkers and overall survival. For all analysis, a two-sided *p* value of < 0.05 was considered statistically significant. *p* values were marked as *<0.05, **<0.01. Given the limited cohort size, statistical comparisons were performed using univariate tests without adjustment for clinical covariates. Thus, survival analysis was interpreted as exploratory and descriptive rather than powered for definitive inferential conclusions. Statistical analysis was performed with R statistical and computing software, version 3.5.0 (http://www.r-project.org), GraphPad Prism, version 10.2.3 (GraphPad, San Diego, CA, USA) and IBM SPSS, version 29.0.

## Results

### Patients’ characteristics and survival

A total of 14 BCLM patients (6 survivors and 8 non-survivors according to the predefined 24-month overall survival cutoff) treated with LRT were included in this exploratory study. Among the 14 patients included, 6 were treated with high-dose rate brachytherapy (HDR-BT) and 8 with selective internal radiation therapy (SIRT). Of the HDR-BT group, 5 patients were classified as survivors and 1 as non-survivor, whereas in the SIRT group, 1 patient was a survivor and 7 were non-survivors. Six patients were treated with HDR-BT (5 survivors and 1 non-survivor), whereas eight patients received SIRT (1 survivor and 7 non-survivors). All patients (median age 68 years, range 38–89, IQR 54–76) had histologically confirmed primary breast cancer. Median OS was 21.9 months (range 3.97–70.77). Tumor classification was performed according to TNM guidelines [34]. Patients who were alive at or beyond the 24-month follow-up were classified as survivors, whereas those who experienced disease-related mortality within this period were classified as non-survivors. Patients’ characteristics are summarized in Supplementary Table 1.

### Longitudinal proteomics analysis of plasma samples differentiates survivors from non-survivors

**Fig. 1:**
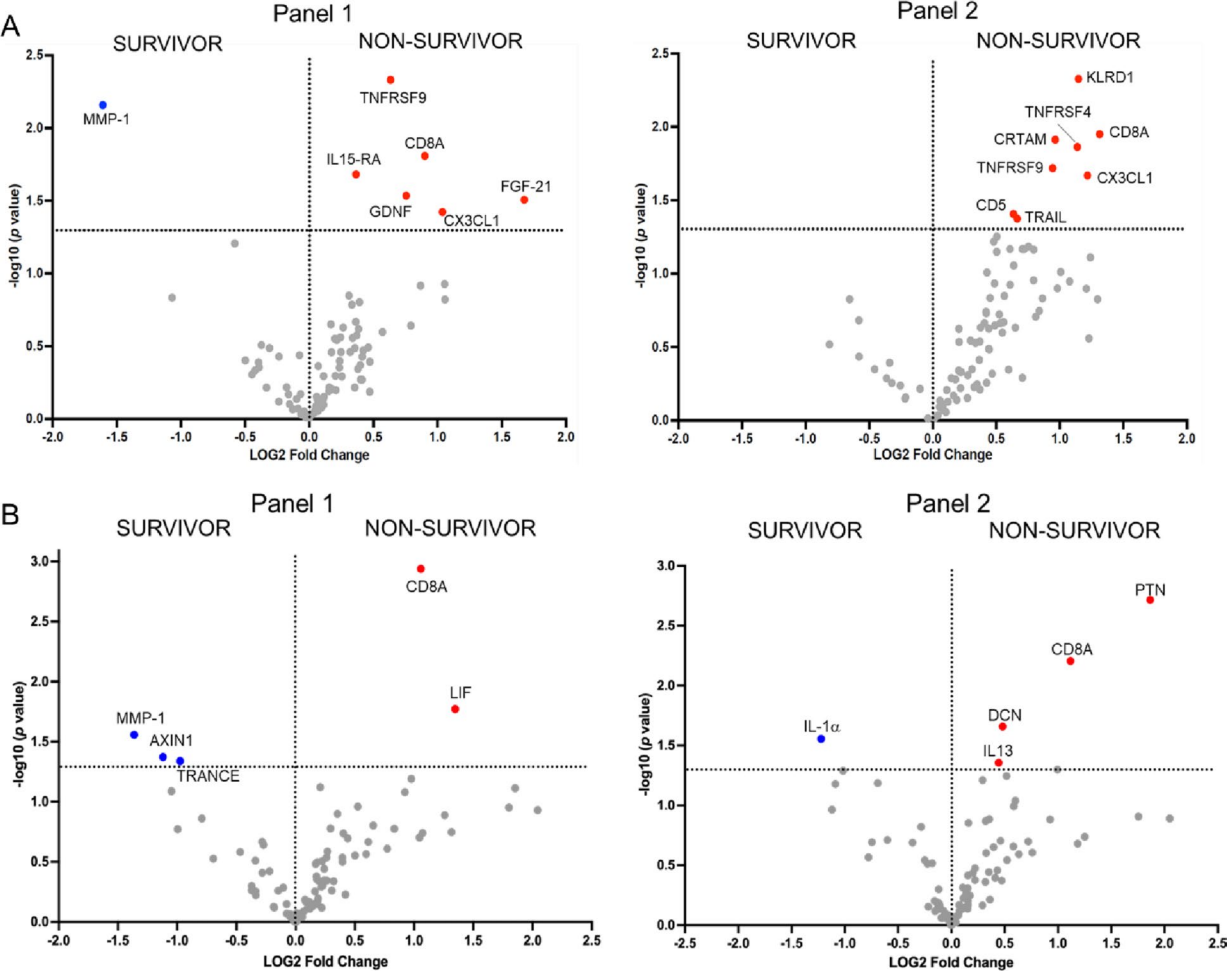
Differentially expressed plasma proteins in survivors and non-survivors before and after therapy and correlation with survival. (**A**) Volcano plot depicting differentially expressed proteins at baseline comparing survivors and non-survivors. Panel 1 includes inflammatory proteins, Panel 2 immune-oncology related proteins. Red dots indicate proteins upregulated in non-survivors; blue dots indicate proteins upregulated in survivors. The horizontal dotted line indicates the significance threshold of −log10(*p* value) = 1.301, corresponding to a *p*-value of 0.05. (**B**) Volcano plot showing differentially expressed proteins after therapy between survivors and non-survivors. Panels, color coding and threshold as in panel A

Plasma samples collected both before and after LRT were analyzed using the Proximity Extension Assay (PEA) in 14 patients (6 survivors and 8 non-survivors) to compare systemic proteomic profiles associated with clinical outcome. Two Olink Target 96 panels including inflammatory markers (Panel 1) and immune-oncology–related markers (Panel 2), for a total of 192 analytes (for the complete protein list, Supplementary Table 2) were used. An unpaired differential expression analysis was initially conducted on baseline (pre-treatment) plasma samples to compare survivors and non-survivors (Fig. 1A). A total of twelve proteins were found to differ significantly between survivors and non-survivors (all *p* < 0.05). Eleven proteins were significantly elevated in non-survivors: IL-15RA (a cytokine receptor involved in T and NK cell activation, *p* = 0.02, Log₂FC = 1.28), TNFRSF4 and TNFRSF9 (co-stimulatory receptors enhancing T-cell survival and proliferation, *p* = 0.01, Log₂FC = 2.2 and *p* = 0.004, Log₂FC = 1.5, respectively), GDNF (a neurotrophic factor linked to tumor progression, *p* = 0.02, Log₂FC = 1.7), CD8A (a marker of cytotoxic T lymphocytes, *p* = 0.01, Log₂FC = 1.9), CX3CL1 (a chemokine involved in leukocyte adhesion and migration, *p* = 0.03, Log₂FC = 2.0), FGF-21 (a metabolic regulator associated with tumor-associated stress responses, *p* = 0.03, Log₂FC = 3.2), CRTAM and KLRD1 (immune cell markers involved in NK and cytotoxic T cell function, *p* = 0.01, Log₂FC = 1.9 and *p* = 0.004, Log₂FC = 2.2, respectively), CD5 (a regulator of T-cell signaling, *p* = 0.04, Log₂FC = 1.5), and TRAIL (a ligand involved in apoptotic signaling, *p* = 0.04, Log₂FC = 1.6). In contrast, MMP1 (*p* = 0.006, Log₂FC=–1.60) was the only protein significantly elevated in survivors. MMP1 is a matrix metalloproteinase implicated in extracellular matrix remodeling and has been associated with active tissue homeostasis and inflammation. Notably, MMP1 is predominantly secreted by CAFs, key stromal cells that actively reshape the tumor microenvironment and influence immune dynamics and treatment sensitivity. Together, these findings suggest that distinct systemic immune profiles exist prior to therapy. To investigate the potential impact of LRT on systemic proteomic signatures, a similar high-throughput analysis was performed on plasma samples collected after treatment (Fig. 1B). Likewise, this post-therapy assessment aimed to identify treatment-induced changes in circulating protein levels that could differentiate survivors from non-survivors. Among survivors, four proteins remained significantly elevated compared to non-survivors, including MMP1 (*p* = 0.02, Log₂FC=−1.3); Axin1, a regulator of Wnt signaling (*p* = 0.04, Log₂FC=−1.1); TRANCE, associated with immune response modulation (*p* = 0.04, Log₂FC=−0.97) and IL-1α, a pro-inflammatory cytokine implicated in immune activation (*p* = 0.03, Log₂FC = 0.4). Conversely, in non-survivors, elevated levels of five immune and stromal-related proteins were observed, including CD8A, a marker of cytotoxic T cells (*p* = 0.001, Log₂FC = 2.1); LIF, a cytokine involved in immune regulation and tumor progression (*p* = 0.01, Log₂FC = 2.5); IL-13, associated with Th2 immune responses (*p* = 0.04, Log₂FC = 1.3); DCN, an extracellular matrix protein with known roles in tumor-stroma interactions (*p* = 0.02, Log₂FC = 1.4) and PTN, a growth factor involved in angiogenesis and metastasis (*p* = 0.001, Log₂FC = 3.6). These findings suggest that distinct systemic proteomic profiles persist or emerge following therapy, reflecting divergent biological pathways activated in survivors versus non-survivors.

**Fig. 2:**
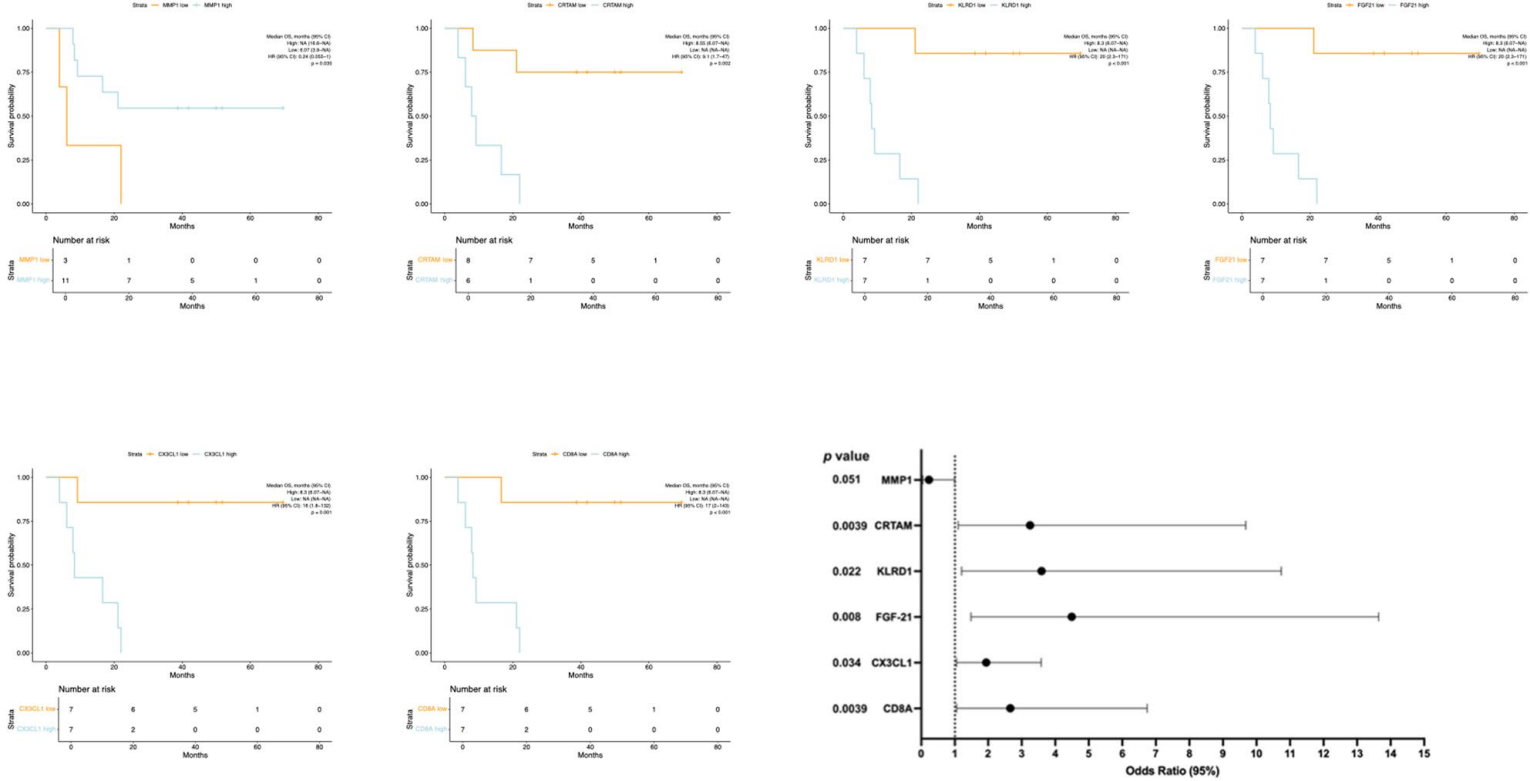
Differentially expressed plasma proteins and correlation with survival. (**A**) Kaplan-Meier curves depicting overall survival stratified by baseline levels and (**B**) univariate Cox regression analysis of 6 plasma proteins significantly associated with survival. Cut-offs were determined using ROC analysis (blue line: high; orange line: low). Log-rank *p*-values and AUC values are reported for each protein

Kaplan–Meier survival analysis suggested that elevated baseline levels of several proteins, including CRTAM, KLRD1, FGF-21, CXCL1 and CD8A (Fig. 2A), CX3CL1, TNFRSF4, TNFRSF9, IL-15RA, GDNF (data not shown) were significantly associated with shorter overall survival (OS) (all *p* < 0.05). These proteins demonstrated strong prognostic performance, each with an area under the curve (AUC) exceeding 0.8, indicating high discriminatory power. By contrast, higher circulating levels of MMP1 were significantly correlated with prolonged OS (*p* < 0.05, AUC > 0.8). Cox proportional hazards regression analysis showed that only MMP1, CRTAM, KLRD1, FGF-21, CX3CL1 and CD8A were independently associated with OS (all *p* < 0.05), suggesting a potential association with survival outcomes (Fig. 2B). While CRTAM, KLRD1, and CD8A are proteins linked to the immune response, MMP1, FGF-21, and CX3CL1 are markers linked to CAFs and epithelial–mesenchymal transition (EMT).

### Immune cell infiltration in metastatic breast cancer patients and survival

**Fig. 3:**
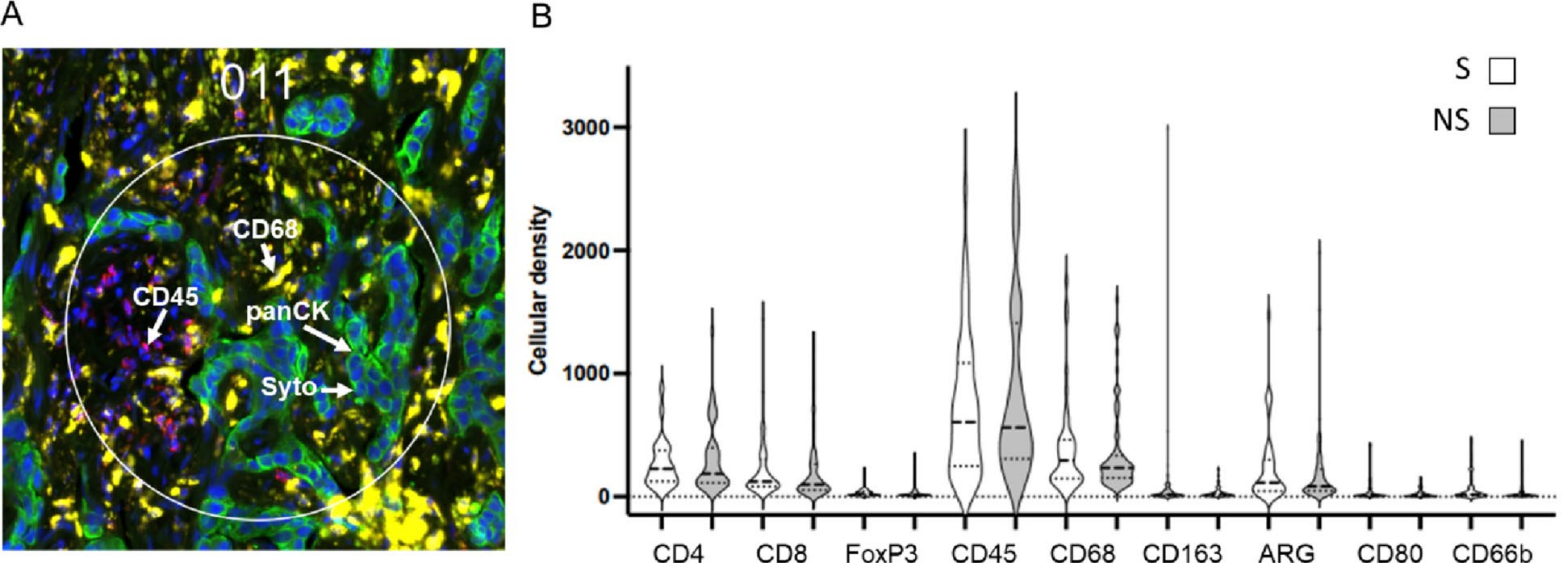
Immune cell infiltration by therapy response in BCLM. (**A**) Region of interest (ROI), selected to represent the tumor and the adjacent tissue areas. (**B**) Distribution of a representative range of immune cells in tumors of survivors (S) and non-survivors (NS) without distinguishing between epithelial compartment (defined as panCK+) and immune stroma (IS) (defined as CD45 + and CD68+). Mann Whitney U-test revealed no significant differences between the groups (all *p* > 0.05)

To validate and spatially contextualize the proteomic alterations observed in plasma, we employed DSP on tumor tissues to examine the localization and expression of relevant markers. First, we investigated the distribution of a representative range of immune cells (CD4 + helper T cell, CD8 + cytotoxic T cell, FoxP3 + regulatory T cell, CD68 + macrophage, CD163 + and ARG1 + M2 macrophage, CD80 + M1 macrophage, CD66b+ neutrophil) in tumors of survivors and non-survivors collected at baseline, without distinguishing between epithelial compartment (defined as panCK+) and immune stroma (IS) (defined as CD45 + and CD68+) [35]. The quantification of the different immune cell types in itself did not show any significant difference (all *p* > 0.05) between survivors and non-survivors (Fig. 3).

### ROIs selection and protein quantification in separate compartments

**Fig. 4:**
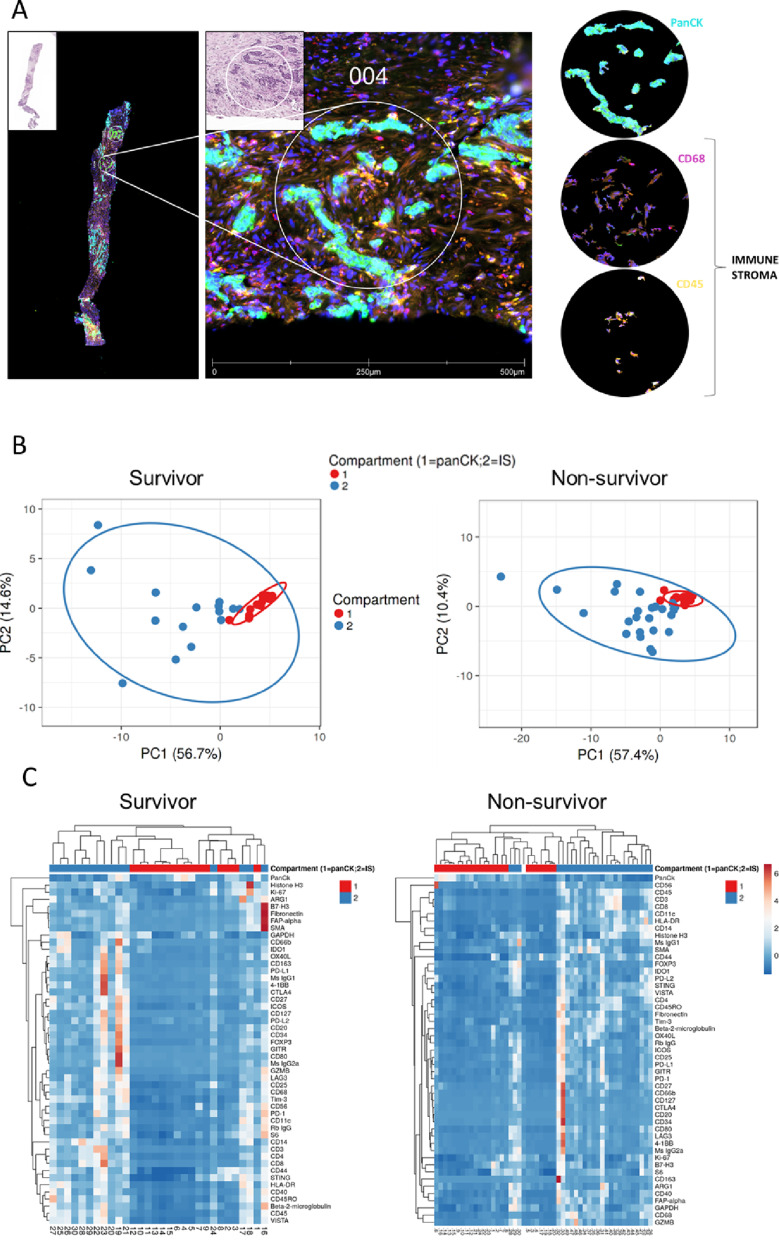
Digital spatial profiling of protein expression to compare tumor and adjacent tissue in BCLM. (**A**) Regions of interest (ROI) were selected to represent the tumor and the adjacent tissue areas. Tissues were stained with Syto13 (blue), anti-PanCK (cyan), anti-CD68 (purple) and anti-CD45 (yellow). (**B**) PCA distribution of all segments in BCLM and healthy tissues. (**C**) Heatmap showing scaled protein counts for all segment types in the dataset.

To further dissect spatially distinct immune and stromal features associated with treatment response, we next performed DSP to compare specific compartments within the tumor microenvironment. A total of 40 distinct regions of interest (ROIs) (i.e., areas of non-necrotic tumor), were defined based on spatial enrichment of key cellular morphology markers: pan-Cytokeratin (PanCK) for epithelial/tumor cells, CD68 for macrophages, and CD45 for general leukocyte populations, in both tumor and adjacent healthy tissues (Fig. 4 A). For each compartment, AOIs (*n* = 120) were selected and analyzed by means of a 49-plex panel characterizing immune cell typing, immune activation status, myeloid status and identifying druggable immune-oncology protein targets (Table 1). PCA and unsupervised hierarchical clustering showed segregation between the IS and PanCK compartments. As expected, the IS compartment exhibited overall higher expression levels of immune-related proteins, reflecting increased immune cell density and activity, thereby validating the accuracy of the segmentation (Fig. 4B C).

### Protein distribution in the epithelial and immune-stroma compartments

**Fig. 5:**
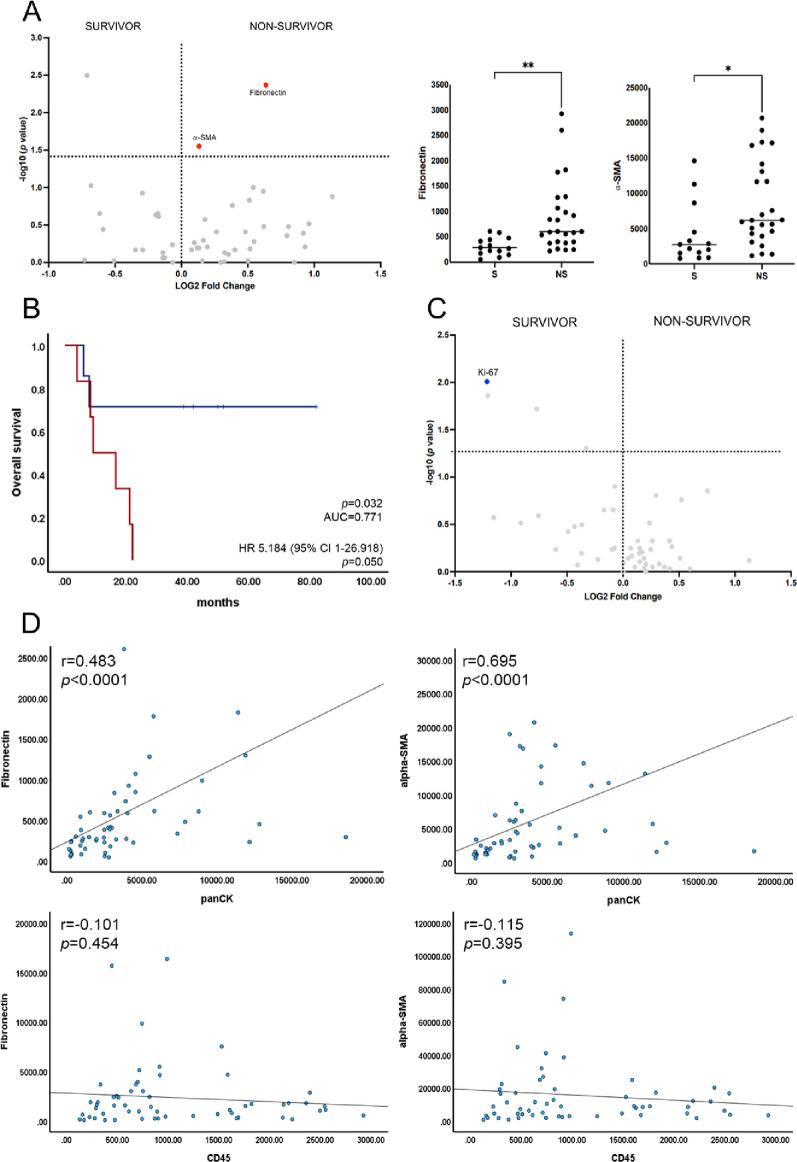
Compartment-specific protein expression and association with clinical outcome. (**A**) Volcano plot (left panel) depicting differentially expressed proteins across the epithelial compartments between survivors and non-survivors. Red dots represent proteins expressed at higher levels in non-survivors, blue dots represent proteins expressed at higher levels in survivors. The horizontal dotted line indicates the significance threshold of −log10(*p*) = 1.301, corresponding to a *p*-value of 0.05. Differences in the expression levels of Fibronectin and α-SMA were confirmed by Mann Whitney U-test (right panel). (**B**) Kaplan-Meier curves depicting overall survival stratified by baseline levels and univariate Cox regression analysis of Fibronectin detected in the epithelial compartment. Patients with a higher concentration of fibronectin (red line) had a worse clinical outcome (*p* = 0.032) with a HR = 5.184 (*p* = 0.050). Cut-off was determined using ROC analysis. Log-rank p-values and AUC values are reported. (**C**) Volcano plot depicting differentially expressed proteins across the IS compartment between survivors and non-survivors. Blue dots represent proteins expressed at higher levels in survivors. The horizontal dotted line indicates the significance threshold of −log10(*p*) = 1.301, corresponding to a *p*-value of 0.05. (**D**) Pearson correlation analysis between fibronectin/α-SMA and panCK (upper panels) and CD45 (lower panel). Each dot represents an individual sample

To determine whether the systemic fibroblast-associated signatures identified in plasma could be traced to specific tumor compartments, we assessed whether spatially resolved proteomic features were associated with treatment response by analyzing epithelial and immune-stromal segments separately. In the epithelial compartment of non-survivors, Fibronectin (*p* = 0.0042) and α-SMA (*p* = 0.028) were significantly upregulated (Fig. 5A). Fibronectin detected in the epithelial compartment was associated with a shorter OS (*p* = 0.032, AUC = 0.771) and a HR = 5.184 (*p* = 0.050) (Fig. 5B). Fibronectin expression was absent in the immune compartment (all *p* > 0.05), which was characterized by a higher level of Ki-67 (*p* = 0.0098) (Fig. 5C). The localization of Fibronectin and α-SMA in the epithelial compartment was supported by their association with CK expression (*response*:0.483, *p* < 0.0001 and *response*:0.695, *p* < 0.0001, respectively) and a non-significant association with the CD45 expression (*response*: −0.101, *p* = 0.454 and *response*: −0.115 and *p* = 0.395, respectively) (Fig. 5D). Neither α-SMA nor Ki-67 showed any association with clinical outcome (all *p* > 0.05, data not shown). Together, these findings indicate that fibroblast-associated proteins are predominantly enriched within the epithelial tumor compartment in non-survivors, suggesting that local fibroblast-driven remodeling of the tumor microenvironment may underlie the systemic proteomic signatures observed in plasma.

### Expression of MMP1 in tumor as a marker of CAFs

**Fig. 6:**
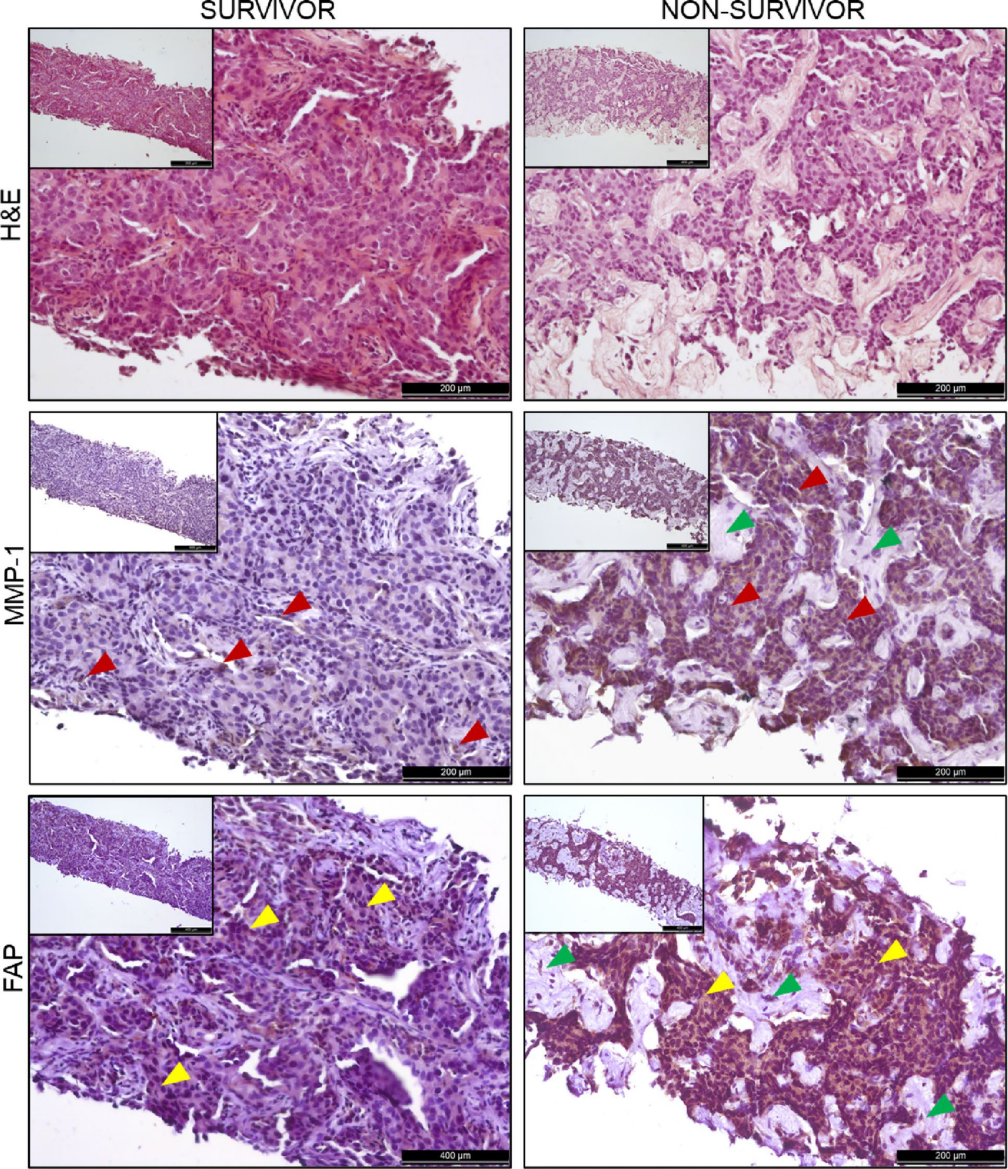
Representative images of H&E, MMP1 and FAP staining in tumor tissues from survivors (left panel) and non-survivors (right panel) before treatment. Red arrows indicate MMP1 positive cells, which in survivors correspond only to endothelial cells and in non-survivors also to tumor cells. Yellow arrows indicate FAP positive tumoral cells. Green arrows indicate stromal areas. Magnification: 10X, scale bar: 400 μm; 20X, scale bar: 200 μm

Plasma proteomics analysis revealed that patients with longer overall survival (OS) exhibited elevated baseline levels of MMP1, suggesting a potential prognostic role for this protein. MMP1 is a key effector molecule secreted by CAFs in various tumor types, including breast cancer [3]. Notably, in non-survivors, we observed high intratumoral expression of α-SMA and fibronectin, two canonical CAFs’ markers. This led us to hypothesize that plasma MMP1 levels may inversely reflect its expression within the actual tumor tissue itself, potentially due to differences in CAF abundance or activity between patient groups. To explore this, we conducted immunohistochemical analysis of MMP1 in tumor samples from a representative subset of patients, including both survivors and non-survivors. As anticipated, tumoral MMP1 expression was markedly elevated in non-survivors, being evident in the tumoral areas and not in the stroma. On the contrary, survivors showed minimal or negligible staining in endothelial cells. In similar tissue compartments, non-survivors demonstrated higher expression of fibroblast activation protein (FAP), another marker of CAFs, supporting the hypothesis that MMP1 is predominantly released by these cells. These observations raise the possibility that systemic MMP1 levels may reflect distinct biological contexts compared to local tumor expression. However, the mechanisms underlying this apparent discrepancy remain to be elucidated (Fig. 6).

### Correlation between Fibronectin/α-SMA expression in the epithelial compartment and fibroblast-related systemic cytokines

**Fig. 7:**
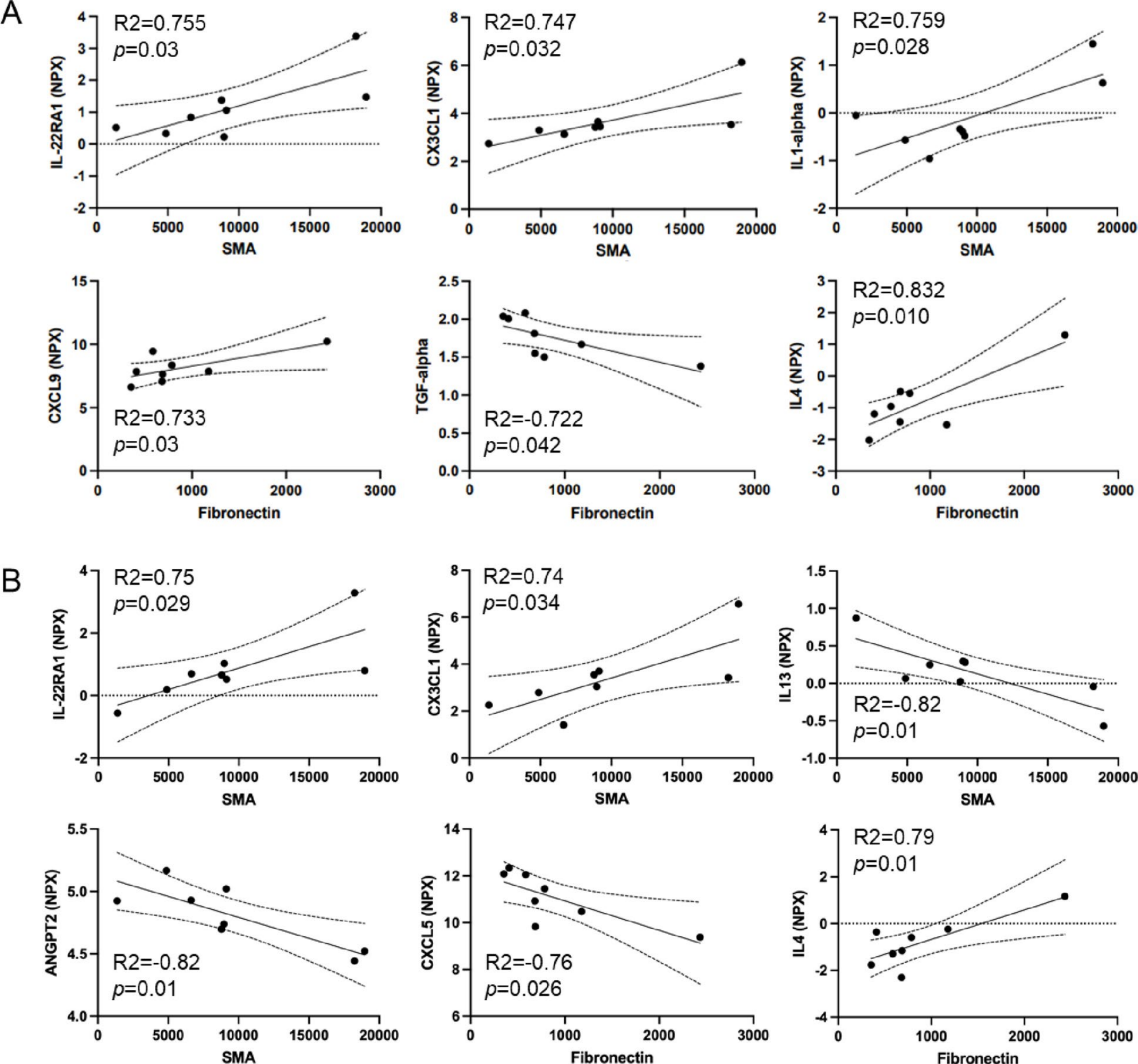
Correlation between epithelial Fibronectin/α-SMA expression and systemic fibroblast-related cytokines. (**A**) Pearson correlation analysis between Fibronectin or α-SMA expression in the epithelial compartment and circulating cytokine levels measured before LRT. (**B**) Pearson correlation analysis between Fibronectin or α-SMA expression and circulating cytokine levels measured after LRT. Each dot represents an individual sample

To determine whether localized Fibronectin/α-SMA expression within the tumor microenvironment reflects broader, systemic cytokine signatures associated with CAF activity, we correlated between local expression levels of Fibronectin/α-SMA in the epithelial compartment and circulating cytokine concentrations. At baseline, six CAF-related cytokines (IL22-RA1, CX3CL1, IL-1α, CXCL9, TGF-α, and IL-4) showed significant positive correlations with Fibronectin or α-SMA levels (all *p* < 0.05, R2 > 0.7) (Fig. 7A). Similarly, following therapy, significant correlations were observed between epithelial Fibronectin/α-SMA expression and post-treatment levels of IL22-RA1, CX3CL1, IL-13, ANGPT2, CXCL5, and IL-4 (all *p* < 0.05, R² >0.7) (Fig. 7B), suggesting a potential association between systemic cytokine profiles and the CAF phenotype over the course of treatment. Given the limited sample size, these correlations should be interpreted with caution and considered in a hypothesis generating manner.

## Discussion

The liver is recognized for its immune-tolerant microenvironment, raising the hypothesis that its surrounding parenchyma may influence how liver metastases respond to radiation [36, 37]. This exploratory study highlights the potential of integrating circulating protein signatures with spatially resolved tumor proteomes to elucidate mechanisms linking systemic biomarkers with tumor microenvironment dynamics. By combining high-throughput plasma proteomics with digital spatial profiling of tumor tissues, we identified distinct immune and stromal features that differentiate survivors from non-survivors following radiation-based locoregional therapy.

Systemic proteomic analysis revealed divergent immune profiles between patients. Prior to treatment, non-survivors exhibited elevated circulating levels of several pro-inflammatory and immune-activating proteins, including CD8A, CX3CL1, and FGF-21. Paradoxically, these markers were associated with shorter overall survival, suggesting a chronically stimulated, yet functionally exhausted immune phenotype, potentially driven by a pro-tumorigenic systemic environment [35, 38, 39]. In contrast, survivors displayed higher plasma levels of matrix metalloproteinase-1 (MMP1), a protein primarily secreted by CAFs [40], which correlated with improved survival. Interestingly, this systemic benefit contrasted with local tumor findings where high intratumoral MMP1 expression, co-localized with FAP+ CAFs, was instead associated to poorer outcomes. These observations highlight an apparent discrepancy between systemic and tumor-associated MMP1 expression. While circulating MMP1 levels were associated with improved survival, increased intratumoral expression was observed in non-survivors, suggesting that systemic and local MMP1 signals may reflect distinct biological contexts within the tumor microenvironment. Several factors may contribute to this difference, including variations in protein secretion dynamics, compartmentalization of stromal signaling, or contributions from non-tumoral tissues to circulating MMP1 levels [37]. Although MMP1 is commonly associated with CAF activity, its cellular origin is not restricted to stromal fibroblasts [41, 42]. Tumor epithelial cells have also been reported to express and secrete MMP family members, including MMP1 [43]. In addition, as MMP1 is a secreted extracellular matrix–remodeling protease, the spatial distribution observed by immunohistochemistry may reflect extracellular protein accumulation rather than the precise cellular site of synthesis. Consequently, the detection of MMP1 within tumor regions may result from secretion by stromal cells, tumor cells, or both, as well as diffusion within the tumor microenvironment. Taken together, these findings should be interpreted cautiously, and further studies using higher-resolution spatial or single-cell approaches will be required to more precisely define the cellular sources and biological roles of MMP1 in this context.

Correlation analysis showed that epithelial fibronectin and α-SMA expression aligned with circulating cytokines such as IL-4, CX3CL1, and IL-13, suggesting a link between systemic cytokine signaling and local fibroblast activation within the tumor microenvironment [44–46]. Given the exploratory design and limited cohort size, the analyses were performed without formal correction for multiple testing. Yet, in small cohorts, correlation coefficients may be unstable and prone to inflation, potentially overestimating the strength of associations. Therefore, the reported associations, including our correlation analyses linking epithelial Fibronectin/α-SMA expression with circulating cytokines, should be interpreted with caution and as hypothesis generating and require validation in larger independent cohorts.

Spatial proteomic profiling revealed that metastatic lesions did not significantly differ in immune marker expression between survivors and non-survivors. The liver harbors a large population of resident macrophages (Kupffer cells), and metastatic niches can recruit additional monocyte-derived macrophages from the circulation [47]. As a result, macrophages often constitute a substantial proportion of the immune infiltrate in liver metastases, including those derived from breast cancer, and may dominate the stromal immune compartment. In addition, macrophages are relatively large cells with abundant cytoplasm and strong CD68 expression, generating a prominent signal in multiplex immunofluorescence imaging. In contrast, many CD45⁺ leukocytes, such as lymphocytes, are smaller and contain less cytoplasm, often exhibiting weaker or more punctate staining patterns. Accordingly, we attribute these morphological and staining features as the likely potential cause for the impression that CD68⁺ cells are more abundant in the images, despite CD45 labeling the broader leukocyte population. However, compartment-specific localization enabled a more precise interpretation of biomarker localization and its clinical significance. Among the most notable findings was the differential expression of Ki-67 (*p* = 0.0098). While epithelial Ki-67 expression did not correlate with survival, its presence in the immune stromal compartment did so, suggesting that immune cell proliferation may be associated with differences in radiosensitivity more than with tumor cell proliferation itself. Equally notable was the compartment-specific localization of fibronectin and α-SMA. Traditionally associated with stromal fibroblasts, these markers were enriched within the epithelial (PanCK+) compartment of non-survivors, with fibronectin serving as an independent predictor of poor outcome. Correlation analysis reinforced this link, showing that epithelial fibronectin and α-SMA expression aligned with circulating cytokines such as IL-4, CX3CL1, and IL-13, suggesting a potential link between systemic cytokine signaling and local fibroblast activation within the tumor microenvironment [44–46]. Given the exploratory design and limited cohort size, the analyses were performed without formal correction for multiple testing. Therefore, the reported associations should be interpreted cautiously and considered hypothesis-generating. Immunocytochemistry further confirmed abundant FAP+ fibroblasts in tumors of non-survivors. The compartment-specific enrichment of fibronectin and α-SMA within the epithelial (PanCK+) compartment of non-survivors raises several possible biological interpretations. One possibility is the infiltration or close spatial interaction of CAFs within tumor epithelial regions, a phenomenon frequently observed in fibrotic tumor microenvironments. Alternatively, tumor cells themselves may undergo epithelial–mesenchymal transition (EMT) or partial epithelial reprogramming, leading to the expression of mesenchymal-associated proteins such as fibronectin and α-SMA. A further explanation may relate to potential technical overlap during compartment segmentation in spatial profiling approaches, where closely intermingled stromal and epithelial regions may lead to partial signal mixing. In addition, as fibronectin is a major extracellular matrix protein, its accumulation in the peritumoral stroma surrounding tumor nests may also contribute to the signal detected within the PanCK+ compartment, particularly in regions with close spatial proximity between epithelial and stromal elements. Importantly, the current analysis does not include lineage-tracing or co-localization markers that would allow definitive attribution of these proteins to specific cellular sources. Therefore, conclusions regarding CAF behavior within epithelial regions should be interpreted with caution and require validation using higher-resolution spatial or single-cell approaches.

From a therapeutic perspective, this dual-layered analysis offers several implications. Baseline plasma proteomic profiling could serve as a non-invasive approach to identify patients unlikely to respond to LRT due to an unfavorable immune-fibrotic signature. Moreover, the spatial localization of fibroblast-associated proteins within epithelial compartments may hold both prognostic and predictive value. In line with preclinical evidences [5, 48], our findings support the rationale for therapeutically targeting fibroblasts in combination with LRT, particularly within fibrotic and immunosuppressive liver metastatic niches [49]. Finally, MMP1 emerges as a potential therapeutic target in BCLM. While early attempts to inhibit MMP1 using broad-spectrum MMP inhibitors have shown high toxicity and lack of efficacy [50], renewed interest has focused on developing selective MMP1 inhibitors and modulating upstream signaling pathways to suppress the expression of MMP1 [51]. Few limitations of this study must be acknowledged. The small cohort size limits statistical power, and the retrospective design introduces potential biases. The distribution of survival outcomes differed between treatment groups, which may represent a potential confounding factor that could not be formally addressed due to the limited sample size. Although the radiation doses applied were functionally equivalent, the delivery modes differed between radioembolization and high-dose rate brachytherapy. Due to the limited number of events in this cohort, Cox regression analyses should be considered exploratory and not sufficient to establish independent prognostic effects. Receiver operating characteristic analyses were performed on the same dataset used to identify candidate biomarkers, and no independent validation cohort was available. Therefore, the reported AUC values may be overestimated due to potential overfitting and should be interpreted as exploratory. In addition, the correlation analyses linking epithelial Fibronectin/α-SMA expression with circulating cytokines should be interpreted with caution. In small cohorts, correlation coefficients may be unstable and prone to inflation, potentially overestimating the strength of associations. Therefore, these observations should be considered hypothesis-generating and require validation in larger independent cohorts.

## Conclusions

Integrating plasma proteomics with spatially resolved tumor profiling provides a promising comprehensive framework for exploring tumor-host interactions in metastatic breast cancer. In this exploratory study, the combined analysis of systemic and spatial proteomic signals highlighted potential biomarkers and microenvironmental features associated with treatment outcome. However, given the limited cohort size, these findings should be interpreted cautiously and considered hypothesis-generating. Future studies in larger, independent cohorts and prospective clinical settings will be required to validate these observations and determine their potential clinical relevance.

## Supplementary Information

Supplementary Material 1

## Data Availability

Data are available from the corresponding author on reasonable request.

## Abbreviations

BC: Breast cancer
BCLM: Breast cancer liver metastases
CAF: Cancer-associated fibroblast
CT: Computed tomography
DSP: Digital spatial profiling
FAP: Fibroblast activation protein
LRT: Locoregional therapy
MMP1: Matrix metalloproteinase 1
MRI: Magnetic resonance imaging
NST: No special type
OS: Overall survival
ROI: Region of interest
TME: Tumor microenvironment
