# Supplementary material for: Integrative plasma-to-spatial proteomics reveals fibroblast-associated signatures in liver metastatic breast cancer
Source: Cancer Cell Int. 2026 Jul 13;26:258. doi: 10.1186/s12935-026-04416-x (PMC13361938; doi:10.1186/s12935-026-04416-x)
Supplement: Supplementary file 1 — Supplementary Material 1 [file 12935_2026_4416_MOESM1_ESM.docx]

**SUPPLEMENTARY MATERIAL**

Supplementary Table 1. Patient characteristics

| Variable | All  n=14 (100%) | Survivor  n=6 (43%) | Non-survivor  n=8 (57%) | *p* |
| --- | --- | --- | --- | --- |
| Age/years |  |  |  | 0.59 |
| Median (range/IQR) | 68 (38-89/54-76) | 66 (38-76/54-75) | 68 (51-89/55-80) |  |
| Gender |  |  |  | - |
| Female | 14 (100%) | 6 (43%) | 8 (57%) |  |
| Subtypes |  |  |  | 0.478 |
| Luminal | 10 (71.4%) | 4 (40%) | 6 (60%) |  |
| HER2 + | 1 (7.1%) | 0 (0%) | 1 (100%) |  |
| Triple negative | 3 (21.5%) | 2 (66.6%) | 1 (33.4%) |  |
| Number of metastases |  |  |  | 0.01 |
| Oligometastatic | 8 (57%) | 6 (75%) | 2 (25%) |  |
| Extensive metastasis | 6 (43%) | 0 (0%) | 6 (100%) |  |
| Cirrhosis |  |  |  | - |
| no | 14 (100%) | 6 (43%) | 8 (57%) |  |
| Extrahepatic metastases |  |  |  | 0.387 |
| Lung | 4 | 1 | 3 |  |
| Bone | 6 | 3 | 3 |  |
| Lymph node | 4 | 0 | 4 |  |
| None | 5 | 2 | 3 |  |
| Tumor histology |  |  |  | - |
| NST | 14 (100%) | 6 (43%) | 8 (57%) |  |
| Tumor grade |  |  |  | 1.00 |
| Differentiated | 0 (0%) | 0 (0%) | 0 (0%) |  |
| Moderately differentiated | 6 (43%) | 3 (50%) | 3 (50%) |  |
| Undifferentiated | 8 (57%) | 3 (37.5%) | 5 (62.5%) |  |

Abbreviations: HER2, human epidermal growth factor receptor 2; NST, no special type

Supplementary Table 2. Olink Target 96 Inflammation panel (Panel 1)

| **Protein Name** | **Abbreviation** |
| --- | --- |
| Adenosine Deaminase | ADA |
| Artemin | ARTN |
| Axin-1 | AXIN1 |
| Beta-nerve growth factor | Beta-NGF |
| Caspase-8 | CASP-8 |
| C-C motif chemokine 3 | CCL3 |
| C-C motif chemokine 4 | CCL4 |
| C-C motif chemokine 19 | CCL19 |
| C-C motif chemokine 20 | CCL20 |
| C-C motif chemokine 23 | CCL23 |
| C-C motif chemokine 25 | CCL25 |
| C-C motif chemokine 28 | CCL28 |
| CD40L receptor | CD40 |
| CUB domain-containing protein 1 | CDCP1 |
| C-X-C motif chemokine 1 | CXCL1 |
| C-X-C motif chemokine 5 | CXCL5 |
| C-X-C motif chemokine 6 | CXCL6 |
| C-X-C motif chemokine 9 | CXCL9 |
| C-X-C motif chemokine 10 | CXCL10 |
| C-X-C motif chemokine 11 | CXCL11 |
| Cystatin D | CST5 |
| Delta and Notch-like epidermal growth factor-related receptor | DNER |
| Eotaxin | CCL11 |
| Eukaryotic translation initiation factor 4E-binding protein 1 | 4E-BP1 |
| Fibroblast growth factor 21 | FGF-21 |
| Fibroblast growth factor 23 | FGF-23 |
| Fibroblast growth factor 5 | FGF-5 |
| Fibroblast growth factor 19 | FGF-19 |
| Fms-related tyrosine kinase 3 ligand | Flt3L |
| Fractalkine | CX3CL1 |
| Glial cell line-derived neurotrophic factor | GDNF |
| Hepatocyte growth factor | HGF |
| Interferon gamma | IFN-gamma |
| Interleukin-1 alpha | IL-1 alpha |
| Interleukin-2 | IL-2 |
| Interleukin-2 receptor subunit beta | IL-2RB |
| Interleukin-4 | IL-4 |
| Interleukin-5 | IL5 |
| Interleukin-6 | IL6 |
| Interleukin-7 | IL-7 |
| Interleukin-8 | IL-8 |
| Interleukin-10 | IL10 |
| Interleukin-10 receptor subunit alpha | IL-10RA |
| Interleukin-10 receptor subunit beta | IL-10RB |
| Interleukin-12 subunit beta | IL-12B |
| Interleukin-13 | IL-13 |
| Interleukin-15 receptor subunit alpha | IL-15RA |
| Interleukin-17A | IL-17A |
| Interleukin-17C | IL-17C |
| Interleukin-18 | IL-18 |
| Interleukin-18 receptor 1 | IL-18R1 |
| Interleukin-20 | IL-20 |
| Interleukin-20 receptor subunit alpha | IL-20RA |
| Interleukin-22 receptor subunit alpha-1 | IL-22 RA1 |
| Interleukin-24 | IL-24 |
| Interleukin-33 | IL-33 |
| Latency-associated peptide transforming growth factor beta-1 | LAP TGF-beta-1 |
| Leukemia inhibitory factor | LIF |
| Leukemia inhibitory factor receptor | LIF-R |
| Macrophage colony-stimulating factor 1 | CSF-1 |
| Matrix metalloproteinase-1 | MMP-1 |
| Matrix metalloproteinase-10 | MMP-10 |
| Monocyte chemotactic protein 1 | MCP-1 |
| Monocyte chemotactic protein 2 | MCP-2 |
| Monocyte chemotactic protein 3 | MCP-3 |
| Monocyte chemotactic protein 4 | MCP-4 |
| Natural killer cell receptor 2B4 | CD244 |
| Neurotrophin-3 | NT-3 |
| Neurturin | NRTN |
| Oncostatin-M | OSM |
| Osteoprotegerin | OPG |
| Programmed cell death 1 ligand 1 | PD-L1 |
| Protein S100-A12 | EN-RAGE |
| Signaling lymphocytic activation molecule | SLAMF1 |
| SIR2-like protein 2 | SIRT2 |
| STAM-binding protein | STAMBP |
| Stem cell factor | SCF |
| Sulfotransferase 1A1 | ST1A1 |
| T cell surface glycoprotein CD6 isoform | CD6 |
| T-cell surface glycoprotein CD5 | CD5 |
| T-cell surface glycoprotein CD8 alpha chain | CD8A |
| Thymic stromal lymphopoietin | TSLP |
| TNF-beta | TNFB |
| TNF-related activation-induced cytokine | TRANCE |
| TNF-related apoptosis-inducing ligand | TRAIL |
| Transforming growth factor alpha | TGF-alpha |
| Tumor necrosis factor ligand superfamily member 12 | TWEAK |
| Tumor necrosis factor | TNF |
| Tumor necrosis factor ligand superfamily member 14 | TNFSF14 |
| Tumor necrosis factor receptor superfamily member 9 | TNFRSF9 |
| Urokinase-type plasminogen activator | uPA |
| Vascular endothelial growth factor A | VEGF-A |

Supplementary Table 3. Olink Target 96 Immuno-oncology panel (Panel 2)

| **Protein Name** | **Abbreviation** |
| --- | --- |
| Adenosine deaminase | ADA |
| Adhesion G-protein coupled receptor G1 | ADGRG1 |
| Angiopoietin-1 | ANGPT1 |
| Angiopoietin-1 receptor | TIE2 |
| Angiopoietin-2 | ANGPT2 |
| Arginase-1 | ARG1 |
| Carbonic anhydrase 9 | CAIX |
| Caspase-8 | CASP-8 |
| C-C motif chemokine 13 | MCP-4 |
| C-C motif chemokine 17 | CCL17 |
| C-C motif chemokine 19 | CCL19 |
| C-C motif chemokine 2 | MCP-1 |
| C-C motif chemokine 20 | CCL20 |
| C-C motif chemokine 23 | CCL23 |
| C-C motif chemokine 3 | CCL3 |
| C-C motif chemokine 4 | CCL4 |
| C-C motif chemokine 7 | MCP-3 |
| C-C motif chemokine 8 | MCP-2 |
| CD27 antigen | CD27 |
| CD40 ligand | CD40-L |
| CD40L receptor | CD40 |
| CD70 antigen | CD70 |
| CD83 antigen | CD83 |
| C-X-C motif chemokine 1 | CXCL1 |
| C-X-C motif chemokine 10 | CXCL10 |
| C-X-C motif chemokine 11 | CXCL11 |
| C-X-C motif chemokine 13 | CXCL13 |
| C-X-C motif chemokine 5 | CXCL5 |
| C-X-C motif chemokine 9 | CXCL9 |
| Cytotoxic and regulatory T-cell molecule | CRTAM |
| Decorin | DCN |
| Fibroblast growth factor 2 | FGF2 |
| Fractalkine | CX3CL1 |
| Galectin-1 | Gal-1 |
| Galectin-9 | Gal-9 |
| Granzyme A | GZMA |
| Granzyme B | GZMB |
| Granzyme H | GZMH |
| Heme oxygenase 1 | HO-1 |
| Hepatocyte growth factor | HGF |
| ICOS ligand | ICOSLG |
| Interferon gamma | IFN-gamma |
| Interleukin-1 alpha | IL-1 alpha |
| Interleukin-10 | IL10 |
| Interleukin-12 | IL12 |
| Interleukin-12 receptor subunit beta-1 | IL12RB1 |
| Interleukin-13 | IL13 |
| Interleukin-15 | IL15 |
| Interleukin-18 | IL18 |
| Interleukin-2 | IL2 |
| Interleukin-33 | IL33 |
| Interleukin-4 | IL4 |
| Interleukin-5 | IL5 |
| Interleukin-6 | IL6 |
| Interleukin-7 | IL7 |
| Interleukin-8 | IL8 |
| Killer cell immunoglobulin-like receptor 3DL1 | KIR3DL1 |
| Latency-associated peptide transforming growth factor beta-1 | LAP TGFbeta-1 |
| Lymphocyte activation gene 3 protein | LAG3 |
| Lysosome-associated membrane glycoprotein 3 | LAMP3 |
| Macrophage colony-stimulating factor 1 | CSF-1 |
| Macrophage metalloproteinase-12 | MMP12 |
| Matrix metalloproteinase-7 | MMP7 |
| MHC class I polypeptide-related sequence A/B | MIC-A/B |
| Mucin-16 | MUC-16 |
| Natural cytotoxicity triggering receptor | NCR1 |
| Natural killer cell receptor 2B4 | CD244 |
| Natural killer cells antigen CD94 | KLRD1 |
| Nitric oxide synthase; endothelial | NOS3 |
| Placenta growth factor | PGF |
| Platelet-derived growth factor subunit B | PDGF subunit B |
| Pleiotrophin | PTN |
| Pro-epidermal growth factor | EGF |
| Programmed cell death 1 ligand 1 | PD-L1 |
| Programmed cell death 1 ligand 2 | PD-L2 |
| Programmed cell death protein 1 | PDCD1 |
| Stromal cell-derived factor 1 | CXCL12 |
| T-cell surface glycoprotein CD4 | CD4 |
| T-cell surface glycoprotein CD5 | CD5 |
| T-cell surface glycoprotein CD8 alpha chain | CD8A |
| T-cell-specific surface glycoprotein CD28 | CD28 |
| TNF-related apoptosis-inducing ligand | TRAIL |
| Tumor necrosis factor | TNF |
| Tumor necrosis factor ligand superfamily member 12 | TWEAK |
| Tumor necrosis factor ligand superfamily member 14 | TNFSF14 |
| Tumor necrosis factor ligand superfamily member 6 | FASLG |
| Tumor necrosis factor receptor superfamily member 12A | TNFRSF12A |
| Tumor necrosis factor receptor superfamily member 21 | TNFRSF21 |
| Tumor necrosis factor receptor superfamily member 4 | TNFRSF4 |
| Tumor necrosis factor receptor superfamily member 9 | TNFRSF9 |
| Vascular endothelial growth factor A | VEGFA |
| Vascular endothelial growth factor receptor 2 | VEGFR-2 |
|  |  |
